# Working Women in Large Towns

**Published:** 1890-10-11

**Authors:** 


### WORKING WOMEN IN LARGE TOWNS.

*(Continued from page 389, Vol. VIII.)*

### XII.—ECONOMIC ASPECTS OF THE SUBJECT.

It is obviously impossible to attempt anything in the least approaching an exhaustive survey of our subject, from an economic point of view, without casting to the four winds all consideration of the economy of this journal. But something must be attempted in the way of summing up; some conclusions to which the readers as well as the writer of these articles will have probably found themselves impelled must be stated, and awarded a brief examination. The simplest way will be to enumerate what seem to us to be the principal causes for the terrible distress which prevails among so many of our working women, devoting a few words to each, and finally inquiring whether the remedies which have been or may be suggested are practicable and admissible.

But before entering upon this task, we must just mention one suggested remedy which we dare not include in our list, lest we should be saddled (as Sir Boyle Roche might have put it) with an Irish bull. It runs (here at least our metaphor cannot be challenged) as follows:—Let us help working women by forbidding them to work. Our perplexity, as we ponder this proposition, is something akin to that of the terribly earnest-minded student of Scripture, who, when he read that a Cretan poet had pronounced the Cretans to be "alway liars," observed at once that if the statement were true, then the poet was himself a liar, and therefore it was *not* true; and that on the other hand, if the statement were *not* true, then the poet was not a liar, and therefore it *was* true—getting himself into such a tangle over these terribly conflicting considerations, that he became a hopeless—well, accounts differ as to what exactly he did become, and we will not attempt to pronounce between them.

Seriously, however, we cannot wonder that some people, pondering over the woes of mankind, and seeing that many families could hardly be worse off than they are now if the man were the only bread-winner, should ask whether there is really any benefit in women working at all. They see, again, that where women enter a trade, the result is that the men's wages are reduced, as has lately happened in the cigar factories of East London; and it is well known that men often look upon women-workers as their natural antagonists. Whether, if it were possible to prohibit women's labour all over the world, the result would be in the long run disadvantageous to the labouring class, is a question as tempting to an argumentative mind as it is complicated; but we must sternly confine ourselves to practical politics here, and will only remark that, whatever the result of a world-wide measure might be, the prohibition of female labour in this country alone cannot be thought of. And indeed, looking at the matter as one of internal politics alone, the great preponderance of English women over men must surely forbid such a regulation once and for all. Eight hundred thousand more women than men! How could they support themselves if honest labour was forbidden, except in ways too horrible to contemplate? But if unmarried women are to be allowed to work, we cannot step in and prevent married women from doing the same; and so we must dismiss as hopeless any ideas that legislation can interfere with women taking their place as bread-winners.

At the same time, it is evident that a state of things in which married women are obliged, to any large extent, to contribute to the support of the family, is by no means a satisfactory one; and if a new *régime* could anyhow be inaugurated, under which girls would not only earn a fair wage, but would put by some of it to make their married life more comfortable, we might look forward to a time when married women could really devote themselves to their homes and families, having already done their share of the bread-winning. That such a consummation should be aimed at was strongly urged by Miss Ada Heather-Bigg, in a letter to the *Times*—" The Child, the Girl, and the Woman," which may be still fresh in the remembrance of some of our readers. The very poorest of the Jews, we are told, cherish as sacred the maternity of their women; would it not be well if English people were trained to do the same?

But to return to our promised list of causes of the misery of many of our working-women. Premising that they are but *economical* causes, and even then not an exhaustive list, we will briefly consider each in turn. They are as follows:—Home Work, Working for Pocket-money, Bad Work, Foreign Competition, Foreign Immigration, the Sweating System, and the Small Workshop System.

*Home Work.*—We have had occasion more than once to mention the disadvantages which home-work brings in its train. As we have seen, it is quite an exceptional thing for a home-worker to earn what may be called a good day's wage; and although the low pay of home-workers is no doubt partly owing to the poor work which is all that so many of them can undertake, it is also due to the very fact that they are home-workers. They belong, in fact, to a large and unwieldy body, and a body which may be indefinitely increased by an influx of immigrants, or migrants from the country, or by seasons of depression in the trades of the usual bread-winners. The competition among such workers, then, is always keen, and often well-nigh desperate; and as the employers know this, work is offered, and greedily snatched at, at a wage which will hardly keep body and soul together, even though the unfortunate workers toil from five in the morning till ten at night. And a further result of this is that the wages of indoor workers are seriously reduced. Whether home-workers can ever be taught to combine or not, certain it is that as things are at present, they have no idea of holding together, and making a stand against the wages offered them; and when the indoor hands know that outdoor hands will at once accept work refused by them, how can they resist a reduction in wages? Very often differences of class or of location prevent indoor and outdoor hands from holding any communication with each other; and the employers do their best to add to their difficulties by carefully hindering the sub-contractors who supply home-workers from finding out the scale of payment which prevails in the warehouses. Thus one class is played off against another with deadly effect. When we consider all this, we can hardly wonder that those who have studied the question sometimes even turn over in their minds whether it would be possible to forbid home work altogether. "I suggested [to the Sweating Committee]," says Mr. Walker, "a drastic remedy which would cut at the very root of the sweating system, but I hesitated to recommend it. It was to forbid all home work. This would, however, mean so much hardship in many cases that one would almost tremble at the result."

No; that remedy cannot be tried. But combination, though difficult, is not impossible, even among home workers; and although we fear that their lot may never be a very bright one, any means that we can devise for helping these poor women to help themselves will be well worth a trial.

